# Orbital Inflammation Caused by Aminobisphosphonates

**DOI:** 10.18502/jovr.v17i1.10176

**Published:** 2022-01-21

**Authors:** J Gonzalez Barlatay, C Pagano Boza, GV Hernandez Gauna, JE Premoli

**Affiliations:** ^1^Division of Orbital and Ophthalmic Plastic Surgery, Hospital Italiano de Buenos Aires, Argentina

**Keywords:** Alendronate, Bisphosphonates, Dacryoadenitis, Myositis, Pamidronate, Zoledronate

## Abstract

The aim of this review was to describe orbital inflammation secondary to aminobisphosphonates by analyzing demographic data, clinical presentation, and treatment of the disease. This is a narrative literature review. The search was performed using databases such as Ovid/MEDLINE and COCHRANE. The searches were limited to papers in the English language. We found 43 cases of orbital inflammation due to aminobisphosphonates. Zoledronate was the drug most associated with orbital side effects. Clinical presentation was evident by unilateral involvement (89%), palpebral edema (88%), conjunctival congestion (81%), chemosis (79%), ocular pain (77%), ocular motility impairment (65%), proptosis (56%), and blurred vision (39%). It can affect both eyes (11%) and is accompanied by anterior uveitis (23%). Orbital inflammation secondary to aminobisphosphonates is a severe side effect. Clinically, it cannot be distinguished from idiopathic inflammation of the orbit. Therefore, it is important to rule out previous drug exposure. Timely treatment is vital to expect a favorable outcome, with systemic corticosteroids being the treatment of choice.

##  INTRODUCTION

Bisphosphonate group of drugs are widely used in diseases such as osteoporosis, Paget's disease, osteoclastic bone metastases, and multiple myeloma. These drugs have a high affinity for bone tissue, where they combine with hydroxyapatite crystals to inhibit bone resorption. As a result, untoward bone events decrease, and pain is relieved. Among the well-known adverse effects, the possibility of triggering an acute systemic inflammatory phase response, characterized by fever, pain, nausea, and fatigue within the first 72 hr after administration occurs in approximately 40–50% of the patients.^[1]^ Symptoms are usually transient and resolve spontaneously. However, in several cases, they may be treated with analgesic and antipyretic drugs. The clinical presentation is accompanied by a decrease in the lymphocyte count and an increase in pro-inflammatory markers, such as IL-6, IFN-γ, and TNF-α.^[2,3,4,5]^

Ocular adverse effects related to bisphosphonates have also been reported. The most frequent complications are conjunctivitis, anterior uveitis, episcleritis, and scleritis.^[6]^ Orbital inflammation indicates its clinical severity. It can range from minimal congestion to severe inflammation with visual impairment if not promptly diagnosed and treated in time. Clinically, it cannot be differentiated from idiopathic orbital inflammation. The aim of this study was to perform a literature review on orbital inflammation secondary to bisphosphonates to increase the knowledge of rare adverse effects and determine the best management methods.

##  METHODS

The search was performed using databases such as Ovid/MEDLINE and COCHRANE, using English language restriction in the electronic searches for papers. We searched electronic databases in August 2020. The keywords used for the search were: bisphosphonates, OR orbital inflammation, OR myositis, OR ocular side effects, OR ocular adverse effects, and OR ocular inflammation. At the same time, the search was performed by changing the word bisphosphonate with aminobiphosphonates, zoledronate, ibandronate, alendronate, and pamidronate.

### Selection Criteria

All the papers that described orbital inflammation due to aminobisphosphonates were included. Patients with intraocular side effects that did not involve the orbit were excluded. A database with demographic data, type of drug, disease onset time, clinical characteristics, and treatment of choice was created.

The study was approved by the Institutional Review Board at the *Instituto Universitario del Hospital Italiano de Buenos Aires* and adhered to the tenets of the Declaration of Helsinki.

##  RESULTS

A total of 43 cases of orbital inflammation due to aminobisphosphonates were found in 26 articles published in Ovid/MEDLINE and COCHRANE, including case reports and reviews.^[7,8,9,10,11,12,13,14,15,16,17,18,19,20,21,22,23,24,25,26,27,28,29,30,31,32,33]^ The first article on this topic was published in 1999^[7]^ and the last one in 2019.^[33]^ Demographic data of the patients are summarized in Table 1. Zoledronate was the drug most associated with orbital side effects. The clinical presentations are summarized in Table 2. Unilateral involvement occurred in 89% of the patients. Symptoms and signs included palpebral edema (88%), conjunctival congestion (81%), chemosis (79%), ocular pain (77%), ocular motility impairment (65%), proptosis (56%), and blurred vision (39%). Only two cases had complications, one had a severe reduction in visual acuity due to anterior ischemic optic neuritis (AION),^[13]^ and other reported recurrent orbital inflammation without visual impairment.^[30]^

**Table 1:** Patients' demographic data


Total patients	43
Age (yr), mean ± SD	65.39 ± 9.1
Sex	Female 60% (*n* = 26)
	Male 40% (*n* = 17)
Reason for aminobisphosphonates use	Osteoporosis 56% (*n* = 24)
	Metastasis 21% (*n* = 9)
Type of aminobisphosphonates	Zoledronate 67% (*n* = 29)
	Alendronate 14% (*n* = 6)
	Pamidronate 12% (*n* = 5)
	Risedronate 7% (*n* = 3)
SD, standard deviation	

**Table 2:** Clinical presentation and treatment


Clinical presentation	Unilateral 89% (*n* = 38)
	Palpebral edema 88% (*n* = 38)
	Conjunctival congestion 81% (*n* = 35)
	Chemosis 79% (*n* = 34)
	Ocular pain 77% (*n* = 33)
	Motility impairment 65% (*n* = 28)
	Proptosis 56% (*n* = 24)
	Blurred vision 39% (*n* = 17)
Complications	AION 2.32% (*n* = 1)
Type of treatment	Systemic corticoid 72% (*n* = 31)
	Oral Prednisolone alone 48.83% (*n* = 21)
	Methylprednisolone EV + Oral Prednisolone 23.25% (*n* = 10)
	Solve spontaneously 11.63% (*n* = 5)
	Without data 9.30 (*n* = 4)
	NSAIDs 4.65 % (*n* = 2)
	Topic Prednisolone 2.23% (*n* = 1)
AION, anterior ischemic optic neuritis; NSAIDs, nonsteroids anti-inflammatory drugs	

A total of 27 patients stopped treatment with bisphosphonates due to orbital inflammation, three patients continued treatment despite orbital involvement, and no severe complications were reported.

##  DISCUSSION

Orbital inflammation caused by bisphosphonates is a rare adverse drug reaction. To date, only 43 case reports have been published worldwide.^[7,8,9,10,11,12,13,14,15,16,17,18,19,20,21,22,23,24,25,26,27,28,29,30,31,32,33]^ The route of administration seems to be associated with latency time for the onset of symptoms. The patients treated with oral alendronate started showing signs and symptoms between 15 and 21 days after treatment, while the patients treated with intravenous pamidronate and zoledronate presented them 3 days later. Zoledronate is the bisphosphonate most frequently associated with this reaction when compared with others, and it can be related to it being the most frequently used for its effectiveness in the treatment of osteoporosis. The risk of suffering this acute response and its severity is higher after the first intravenous administration and occur less frequently with fewer symptoms in subsequent administrations. The Horizon trial reported an incidence of orbital inflammation associated with the administration of 30% intravenous zoledronate with the first dose, 7% with the second, and 3% with the third dose.^[34]^ Unilateral orbital inflammation was most frequent (89%), but it may be bilateral (11%). Clinical signs and symptoms included palpebral edema (88%), conjunctival congestion (81%), chemosis (79%), ocular pain (77%), ocular motility impairment (65%), proptosis (56%), and blurred vision (39%). Moreover, 23% of the cases were associated with anterior uveitis. On the contrary, this sign may not be associated with idiopathic orbital inflammation; for that reason, when anterior uveitis develops, physicians may exclude bisphosphonate administration. It is typically non-axial, due to the different structures that could be involved, such as lacrimal gland, extraocular muscles, or intraorbital fat, alone or together. The decrease in visual acuity can be multifactorial. Among the causes, we found corneal keratitis, either because of proptosis or lagophthalmos, dacryoadenitis, due to a decrease in the production of tears; and anterior or posterior uveitis is associated with compressive or ischemic optic neuropathy. A 68-year-old male with metastatic prostate cancer was reported to experience severe complications. He consulted the physician two weeks after the onset of ocular pain and redness. He had visual acuity and visual field deficits because of an AION. Ischemia may have been caused by orbital or ocular inflammation contiguously affecting the posterior ciliary arteries that supply the optic disc, creating local small-vessel vasculitis. This highlights the importance of applying the timely treatment once symptoms have started.^[13]^

The mechanism by which these drugs produce inflammation could be related to the presence of a nitrogenous group that rapidly activates monocytes and a subtype of T-cells called gamma-delta, in both *in vitro* ^[35,36,37,38]^ and *in vivo* conditions.^[1,39]^ This activation leads to the release of cytokines and inflammatory mediators that produce an acute inflammatory response. Local inflammation is followed by an acute phase of systemic inflammatory response with the presence of symptoms such as fever, pain, nausea, and fatigue in 25% of the patients. It is worth mentioning that all patients who developed a bilateral orbital condition (11%) also had systemic symptoms.

Currently, oral systemic corticosteroids alone or following an intravenous corticosteroid cycle are the treatment of choice, with excellent results in 72% of the patients.^[31]^ The response was effective in all cases; symptoms and CT scan or MRI findings showed complete resolution when the treatment was started seasonally. Delays in treatment are associated with an increased risk of complications.^[13]^ Only a few cases resolve spontaneously or with nonsteroidal anti-inflammatory drugs (NSAIDs), limiting the current information to suggest this type of therapeutic decision. For this reason, these two options may only be considered in mild inflammation without the risk of visual impairment, or in patients with contraindications to corticosteroid treatment.

It has not yet been proven that the treatment of orbital inflammation requires stopping the use of bisphosphonates. Although most of the studies reported the suspension of the antiresorptive drug as treatment, the three cases published that continued with the bisphosphonate but associated with systemic steroids resolved the orbital inflammation without complications.

Thus, we recommend that in the event of mild symptoms of orbital compromise without risk of visual impairment, bisphosphonate treatment could be continued in conjunction with anti-inflammatory treatment. However, in severe orbital inflammation with visual threat or optic neuropathy, bisphosphonates should be discontinued.

##  SUMMARY

Orbital inflammation caused by aminobisphosphonates is rather infrequent; however, ophthalmologists must recognize this adverse effect secondary to the drug. This condition must be ruled out when orbital inflammation, with or without anterior uveitis is present. Patients' knowledge of the use of these drugs is key to diagnosis. The treatment of choice is the administration of systemic corticosteroids, which are effective in suppressing the inflammatory response with complete resolution when started appropriately. Delayed treatment may be associated with a poor prognosis.

##  Financial Support and Sponsorship

Nil.

##  Conflicts of Interest

There are no conflicts of interest.**

## References

[B1] Olson K, Van Poznak C (2007). Significance and impact of bisphosphonate-induced acute phase
responses. J Oncol Pharm Pract.

[B2] ThiÃ©baud D, Sauty A, Burckhardt P, Leuenberger P, Sitzler L, Green JR, et al (1997). An in vitro and in vivo study of cytokines in the acute-phase response
associated with bisphosphonates. Calcif Tissue Int.

[B3] Buckler HM, Mercer SJ, Hollis S, Richardson P, Anderson D (57). Evaluation of adverse experiences related to pamidronate infusion in
Paget’s disease of bone. Ann Rheum Dis 1998:.

[B4] Dicuonzo G, Vincenzi B, Santini D, Avvisati G, Rocci L, Battistoni F, et al (2003). Fever after zoledronic acid administration is due to increase in
TNF-Î± and IL-6. J Interferon Cytokine Res.

[B5] Reid IR, Gamble GD, Mesenbrink P, Lakatos P, Black DM (2010). Characterization of and risk factors for the acute-phase response
after zoledronic acid. J Clin Endocrinol Metab.

[B6] Macarol V, Fraunfelder FT (1994). Pamidronate disodium and possible ocular adverse drug
reactions. Am J Ophthalmol.

[B7] Mbekeani JN, Slamovits TL, Schwartz BH, Sauer HL (1999). Ocular inflammation associated with alendronate
therapy. Arch Ophthalmol.

[B8] Ryan PJ, Sampath R (2001). Idiopathic orbital inflammation following intravenous
pamidronate. Rheumatology.

[B9] Subramanian PS, Kerrison JB, Calvert PC, Miller NR (2003). Orbital inflammatory disease after pamidronate treatment for
metastatic prostate cancer. Arch Ophthalmol.

[B10] Benderson D, Karakunnel J, Kathuria S, Badros A (2006). Scleritis complicating zoledronic acid infusion. Clin Lymphoma Myeloma.

[B11] Phillips PM, Newman SA (2008). Orbital inflammatory disease after intravenous infusion of zoledronate
for treatment of metastatic renal cell carcinoma. Arch Ophthalmol.

[B12] Sharma NS, Ooi J-L, Masselos K, Hooper MJ, Francis IC (2008). Zoledronic acid infusion and orbital inflammatory
disease. N Engl J Med.

[B13] Seth A, Anderson DP, Albiani DA, Barton JJS (2009). Orbital inflammation and optic neuropathy with zoledronic acid for
metastatic prostate cancer. Can J Ophthalmol.

[B14] Procianoy F, Procianoy E (2010). Orbital inflammatory disease secondary to a single-dose administration
of zoledronic acid for treatment of postmenopausal osteoporosis. Osteoporos Int.

[B15] Yang EB, Birkholz ES, Lee AG

[B16] Missotten G, Verheezen Y (2010). Orbital inflammation after use of zoledronic acid for metastasized
prostate carcinoma. Bull Soc Belge Ophtalmol.

[B17] Yeo J, Jafer AK (2010). Zolendronate associated inflammatory orbital disease. NZ Med J.

[B18] Kaur H, Uy C, Kelly J, Moses AM

[B19] Ortiz-Perez S, Fernandez E, Molina JJ, Sanchez-Dalmau B, Navarro M, Corretger X, et al (2011). Two cases of drug-induced orbital inflammatory disease. Orbit.

[B20] Peterson JD, Bedrossian EH (2012). Bisphosphonate-associated orbital inflammation—a case report and
review. Orbit.

[B21] Rahimy E, Law SK

[B22] Schwab P, Harmon D, Bruno R, Fraunfelder FW, Kim DH (2012). A 55-year-old woman with orbital inflammation. Arthritis Care Res.

[B23] BÃ¶ni C, Kordic H, Chaloupka K (2013). Bisphosphonate-associated orbital inflammatory disease and uveitis
anterior - a case report and review. Klin Monbl Augenheilkd.

[B24] Lefebvre DR, Mandeville JT, Yonekawa Y, Arroyo JG, Torun N, Freitag SK (2014). A case series and review of bisphosphonate-associated orbital
inflammation. Ocul Immunol Inflamm.

[B25] Vora MM, Rodgers IR, Uretsky S

[B26] Pirbhai A, Rajak SN, Goold LA, Cunneen TS, Wilcsek G, Martin P, et al (2015). Bisphosphonate-induced orbital inflammation: a case series and
review. Orbit.

[B27] Gonzalez Barlatay J, Hernandez Gauna G, Premoli J, Luis VR, Jorge PE (2016). Orbital inflammation caused by bisphosphonates case report and
literature review. IJOO.

[B28] Muruganandam M, Sandhu H (2016). Orbital inflammation secondary to zoledronic acid, a rare
presentation. J Clin Rheumatol.

[B29] Umunakwe OC, Herren D, Kim SJ, Kohanim S (2017). Diffuse ocular and orbital inflammation after zoledronate
infusion-case report and review of the literature. Digit J Ophthalmol.

[B30] Tan M, Kalin-Hajdu E, Narayan R, Wong SW, Martin TG (2019). Zoledronic acid-induced orbital inflammation in a patient with
multiple myeloma. J Oncol Pharm Pract.

[B31] Chehade LK, Curragh D, Selva D

[B32] Herrera I, Kam Y, Whittaker TJ, Champion M, Ajlan RS (2019). Bisphosphonate-induced orbital inflammation in a patient on chronic
immunosuppressive therapy. BMC Ophthalmol.

[B33] Keren S, Leibovitch I, Ben Cnaan R, Neudorfer M, Fogel O, Greenman Y, et al

[B34] Black DM, Delmas PD, Eastell R, Reid IR, Boonen S, Cauley JA, et al (2007). Once-yearly zoledronic acid for treatment of postmenopausal
osteoporosis. N Engl J Med.

[B35] Kunzmann V, Bauer E, Feurle J, Weissinger F, Tony HP, Wilhelm M (2000). Stimulation of Î³Î´ T cells by aminobisphosphonates and induction of
antiplasma cell activity in multiple myeloma. Blood.

[B36] Gober H-J, Kistowska M, Angman L, JenÃ¶ P, Mori L, De Libero G (2003). Human T cell receptor Î³Î´ cells recognize endogenous mevalonate
metabolites in tumor cells. J Exp Med.

[B37] Roelofs AJ, Jauhiainen M, MÃ¶nkkÃ¶nen H, Rogers MJ, MÃ¶nkkÃ¶nen J, Thompson K (2009). Peripheral blood monocytes are responsible for Î³Î´ T cell activation
induced by zoledronic acid through accumulation of IPP/DMAPP. Br J Haematology.

[B38] Thompson K, Keech F, McLernon DJ, Vinod K, May RJ, Simpson WG, et al (2011). Fluvastatin does not prevent the acute-phase response to intravenous
zoledronic acid in post-menopausal women. Bone.

[B39] Kunzmann V, Bauer E, Wilhelm M (1999). Î³/Î´ T-cell stimulation by pamidronate. N Engl J Med.

